# Case report: a clinical case of a giant coronary artery aneurysm treated by percutaneous exclusion

**DOI:** 10.1093/ehjcr/ytag190

**Published:** 2026-03-10

**Authors:** Marco D’Amato, David Martí Sánchez, Edurne López Soberon, Matteo Romano, Diego Rodríguez Torres

**Affiliations:** Department of Cardiology, Hospital Central de la Defensa Gómez Ulla, Calle Bolivia, Madrid, Leganés 28918, Spain; Department of Cardiology, Hospital Central de la Defensa Gómez Ulla, Calle Bolivia, Madrid, Leganés 28918, Spain; Department of Cardiology, Hospital Central de la Defensa Gómez Ulla, Calle Bolivia, Madrid, Leganés 28918, Spain; Department of Cardiology, Hospital Central de la Defensa Gómez Ulla, Calle Bolivia, Madrid, Leganés 28918, Spain; Department of Cardiology, Hospital Central de la Defensa Gómez Ulla, Calle Bolivia, Madrid, Leganés 28918, Spain

**Keywords:** giant coronary artery aneurysm, Magnetic resonance imaging, Computed tomography, Paracardiac mass, Case report

## Abstract

**Background:**

This case report contributes to the limited literature on giant coronary artery aneurysms (CAAs), particularly those arising from secondary coronary branches. It highlights the utility of multimodal imaging in diagnosis and supports endovascular management as a viable treatment option in high-risk patients.

**Case summary:**

A middle-aged patient presented with a paracardiac mass incidentally discovered on imaging. Further evaluation revealed a giant aneurysm originating from a secondary branch of the right coronary artery. Given its large size and high rupture risk, the patient underwent successful percutaneous exclusion with a covered stent. Post-procedural recovery was uneventful, and follow-up imaging confirmed aneurysm exclusion without complications.

**Conclusion:**

This case emphasizes the importance of individualized treatment planning for giant CAAs and illustrates the effectiveness of percutaneous intervention using covered stents. It also reinforces the role of comprehensive imaging in guiding both diagnosis and management of rare coronary anomalies.

Learning points

- Multimodality imaging can be used to make an accurate diagnosis and provide management to patients with cardiac/pericardial masses.
- Successful aneurysm exclusion with ultrasound-guided stenting demonstrates the benefits of minimally invasive, personalized multidisciplinary care for high-risk patients.

## Introduction

Pericardial masses are a heterogeneous group of conditions whose accurate characterization is essential, as etiology and prognosis vary widely. The differential diagnosis includes benign, inflammatory, vascular, and neoplastic entities, requiring systematic assessment supported by advanced imaging. In this context, multimodality imaging has become a cornerstone of diagnostic evaluation, with echocardiography, computed tomography, and cardiac magnetic resonance providing complementary information on anatomy, tissue composition, relationship to adjacent structures, and hemodynamic impact.

Vascular lesions are uncommon among pericardial masses. The reported prevalence of giant coronary artery aneurysms (CAAs) ranges from 0.02% to 0.2%. Etiologies include atherosclerosis, Takayasu arteritis, congenital disorders, Kawasaki disease, and percutaneous coronary intervention. They are asymptomatic in most cases. Less common presentations include angina, sudden death, fistula, pericardial tamponade, compression, and heart failure. Clinical complications may include thrombus formation with or without distal embolization, fistula, and rupture^1–5^

Management strategies depend on aneurysm size, location, and symptomatology, ranging from conservative medical therapy to surgical or percutaneous intervention. Appropriate selection of the therapeutic approach is crucial to prevent complications and optimize long-term outcomes.

## Summary figure

**Figure:**
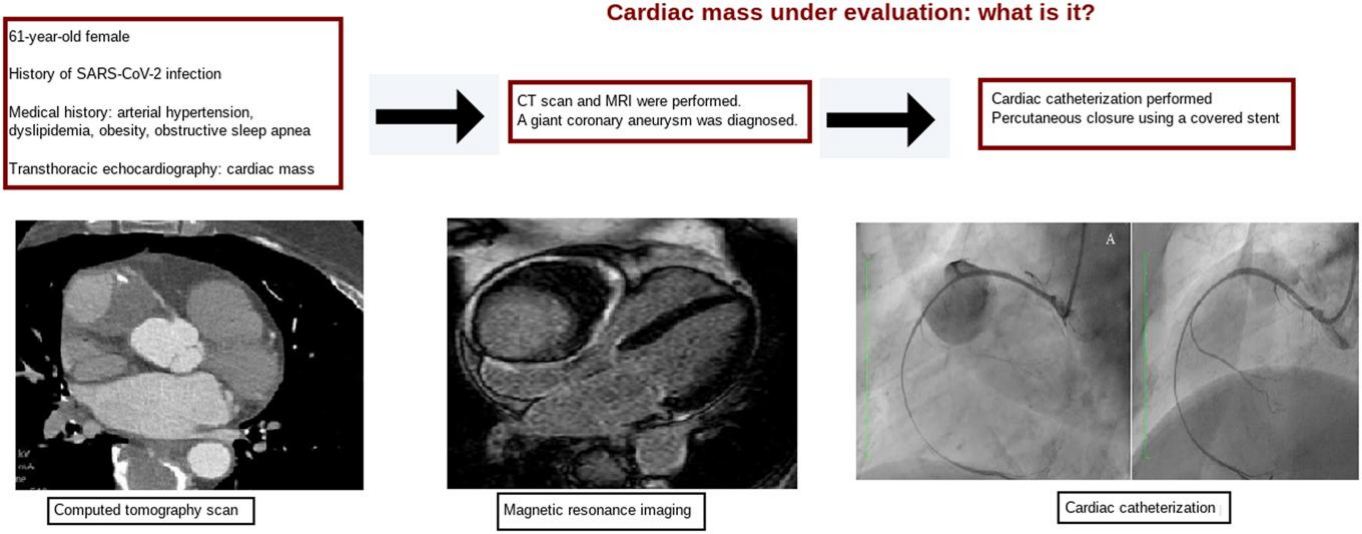


## Case presentation

We report a case of a 61-year-old woman with a past medical history of arterial hypertension, dyslipidemia, obesity, and obstructive sleep apnea. She presented in 2011 and 2019 with episodes of chest pain. These episodes were investigated using SPECT-MIBI, which yielded negative results for myocardial ischemia (*Table 1*).

**Table 1:** Timeline.

Date/year	Clinical event	Description
2011	First episode of chest pain	Stress test clinically negative but electrically positive for ischemia. SPECT-MIBI showed no ischemia
2019	Second episode of chest pain	Oppressive chest pain >1 h. Elevated high-sensitivity troponin T (74.80 ng/l). ECG showed no significant abnormalities. Echocardiogram with normal LVEF. SPECT-MIBI again negative.
2021	SARS-CoV-2 infection	Recovery without major complications.
2022 (outpatient)	Transthoracic echocardiogram	Incidental finding of an immobile pericardial mass in the right coronary sulcus, with heterogeneous content and smooth borders, compressing right heart chambers without tamponade.
2022 (hospital admission)	Mass evaluation	Differential diagnosis included aneurysm, pericardial cyst, hiatal hernia, and pericardial tumors.
2022 (definitive diagnosis)	Advanced imaging (CT, MRI, coronary angiography)	Giant aneurysm (10.4 × 7.7 × 7.4 cm) of the sinoatrial branch of the right coronary artery with partially calcified thrombus. Right coronary artery displaced and narrowed.
2022 (treatment)	IVUS-guided percutaneous covered stent implantation (PK Papyrus® 3/15 mm)	Successful aneurysm exclusion. Dual antiplatelet therapy (aspirin + clopidogrel) initiated.
6 months post-procedure	Follow-up and rehabilitation	Stress test negative for ischemia. Improvement in exertional dyspnea. Coronary CT showed patent stent and effective aneurysm exclusion.
Current status	Clinical condition	Favorable clinical and imaging progression. No symptom recurrence.

In 2022, the patient presented with persistent dyspnea following a COVID-19 infection and an outpatient transthoracic echocardiography was performed. Consequently, she was admitted for comprehensive evaluation and further diagnostic workup of the cardiac mass.

**Figure 1:**
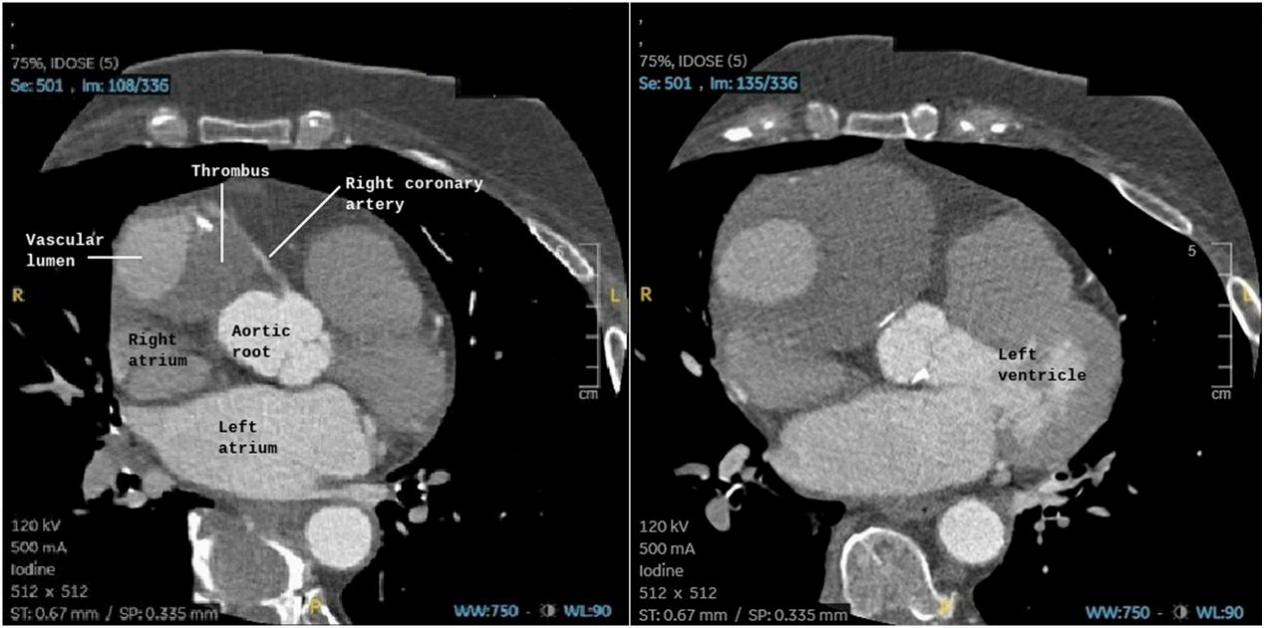
Axial coronary CTA showing a mass with focal contrast enhancement during the arterial phase and a peripheral non-enhancing region suggestive of thrombus along the course of the right coronary artery. The mass partially compresses the right atrium and is consistent with a giant aneurysm.

**Figure 2:**
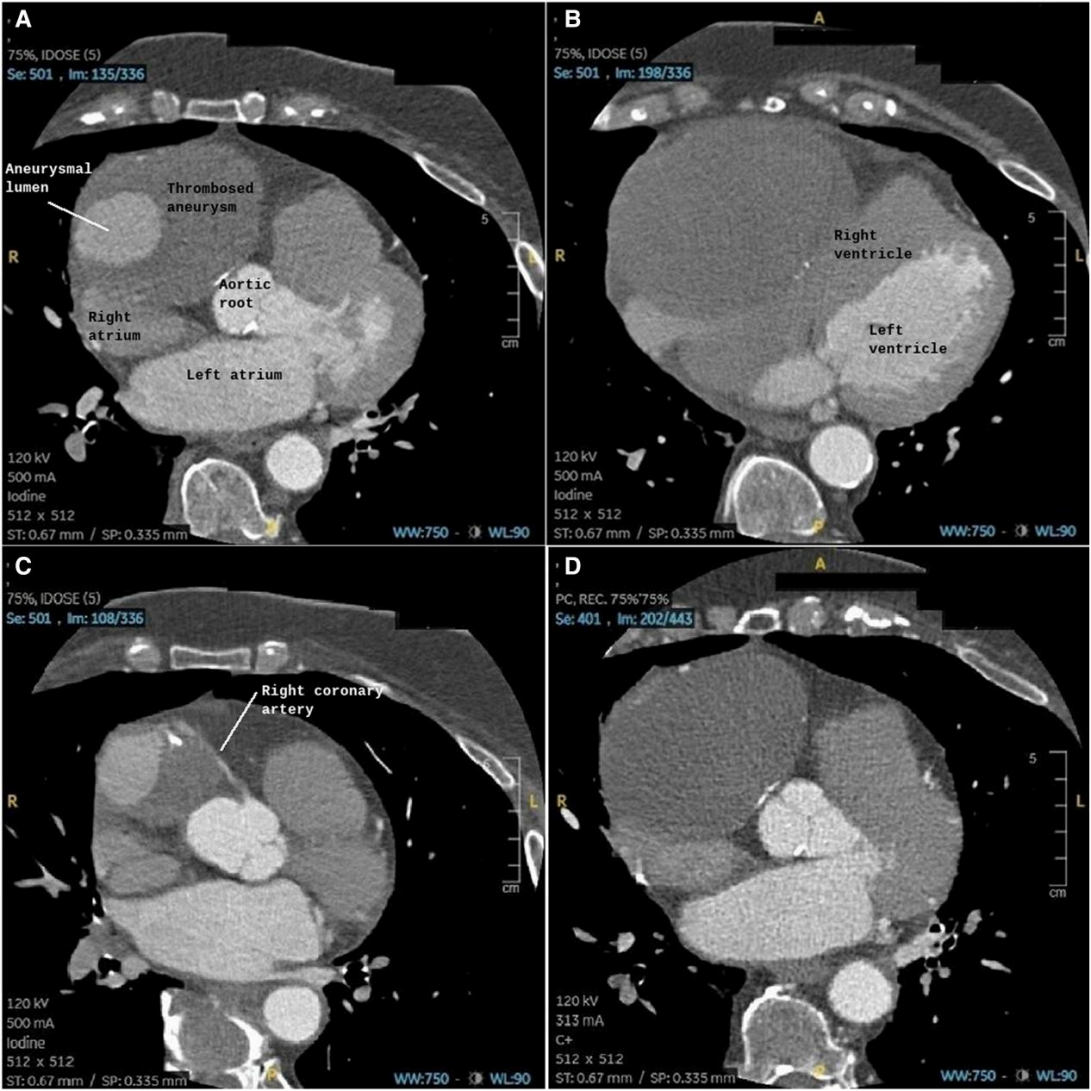
Axial coronary CTA showing a mass adjacent to the right coronary artery, suggestive of a coronary aneurysm, before (*A*) and after (*B*) percutaneous exclusion. Note the absence of contrast enhancement within the mass after exclusion.

**Figure 3:**
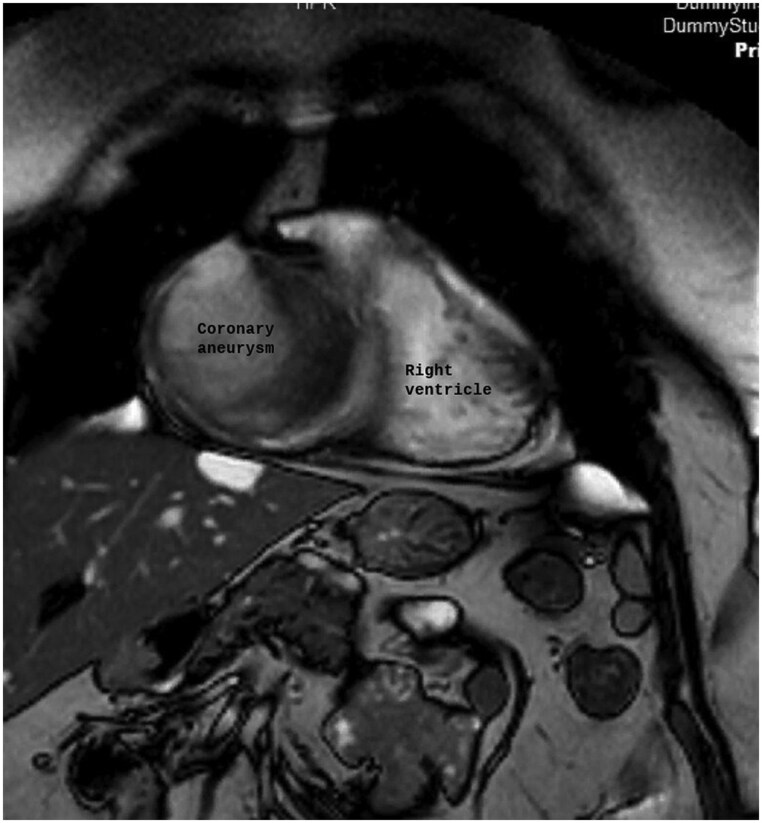
Coronal MRI view showing the right ventricle and, at the anatomical location of the right atrium, a large encapsulated rounded mass consistent with a giant right coronary artery aneurysm.

Transthoracic echocardiography identified a large, well-defined, immobile paracardiac mass in the right coronary sulcus, adjacent to the right atrium, compressing the right heart chambers without signs of cardiac tamponade. CTA was performed due to suspected vascular involvement and revealed a giant aneurysm (10.4 cm × 7.7 cm × 7.4 cm) of the sinoatrial artery branch of the right coronary artery containing a partially calcified thrombus with a partial opacification of the branch. The right coronary artery was displaced anteriorly and exhibited a filiform caliber. Its relationship with neighboring structures was precisely delineated (*Figures 1* and *2*). MRI revealed a heterogeneous signal with areas of thrombus and intralesional contrast filling, ruling out a solid lesion and suggesting a vascular origin (*Figure 3*).

**Figure 4:**
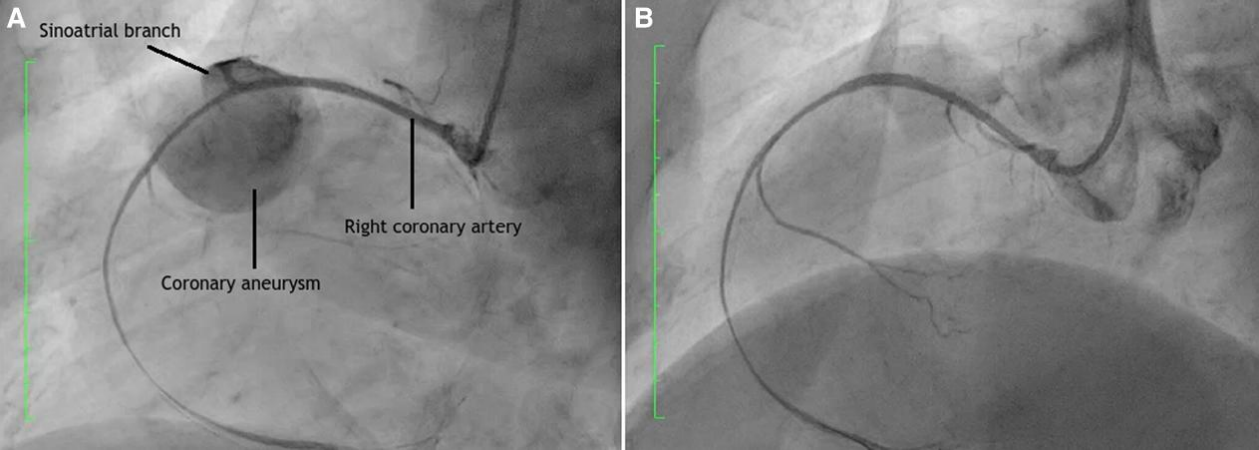
Left anterior oblique projection during coronary artery catheterization showing a giant right coronary artery aneurysm before (*A*) and after percutaneous exclusion.

The case was discussed in a multidisciplinary meeting. Due to the large size of the aneurysm and the associated high risk of rupture, percutaneous treatment was performed. The implantation of a PK Papyrus® covered stent (3/15 mm), guided by intravascular ultrasound (IVUS), was considered the most appropriate strategy because the aneurysm was located in a secondary branch of a nondominant coronary artery (*Figure 4*). Successful exclusion of the aneurysm with a covered stent and favorable follow-up underscores the importance of personalized therapy and structured rehabilitation.^1^ A significant reduction in aneurysm size was not expected due to the presence of the calcified thrombus inside the aneurysm. Pharmacological treatment consisted of dual antiplatelet therapy with acetylsalicylic acid and clopidogrel.

During the 6 months following the percutaneous procedure, she was maintained on dual antiplatelet therapy. She remained asymptomatic, without chest pain or dyspnea. An exercise stress test revealed no evidence of inducible ischemia or stent failure. Follow-up CTA showed a patent stent with proper distal opacification and adequate exclusion of the aneurysm, without signs of endoleak.

## Discussion

This case presents an unusual finding of a giant paracardiac mass. It was detected by outpatient transthoracic echocardiography in a patient with chest pain, whose SPECT-MIBI scan was negative for ischemia. This may be due to the aneurysm’s location in a small, nondominant branch supplying a limited myocardial territory with partially preserved distal perfusion. Additionally, the spatial resolution of SPECT may have contributed to a false-negative result. If the test had been positive, earlier diagnosis and intervention could have reduced the risk of complications by treating the aneurysm sooner.

Multimodal imaging was crucial for accurate diagnosis, differential assessment, and therapeutic decision-making.^2^ Although coronary catheterization angiography remains the gold standard diagnostic technique, CAAs can also be detected using noninvasive tests.

Several studies have shown a correlation between MRI and coronary catheterization angiography in terms of diagnosis of CAAs^6,7^; however, MRI can provide additional information regarding myocardial function, perfusion and scarring.^8^ In addition, coronary catheterization only shows the lumen, whereas MRI allows for the detection of changes in the vessel wall.^9^ CTA can evaluate the coronary tree, CAA size, structure, wall, and lumen characteristics^6,10^; compared with MRI, it is more sensitive for stenoses, and vs. angiography, it is superior for calcifications.

The differential diagnosis of a pericardial mass is broad and includes various causes with differing prognoses. Vascular causes include coronary aneurysms. Pericardial cysts typically show cystic features on imaging. Encapsulated collections, such as effusions or hematomas can mimic masses. Neoplastic causes include primary tumors like mesotheliomas or fibromas, and metastases from lung, breast, or lymphoid cancers. Mediastinal masses, such as lymphadenopathy, anterior mediastinal tumors, or hiatal hernias, can also present as pericardial masses.

The management of coronary aneurysms depends on their size, location, and risk of complications. Surgery is the most common treatment and is indicated for large or high-risk cases. Percutaneous treatment is preferred for aneurysms in secondary branches or in patients with high surgical risk, although long-term outcomes remain unknown. Medical therapy is reserved for small or asymptomatic aneurysms^8,11,12^

In our patient, coronary catheterization with percutaneous exclusion and dual antiplatelet therapy was performed. Covered stent exclusion has high revascularization rates in major arteries. Here, exclusion in a secondary vessel yielded a favorable outcome. IVUS-guided implantation ensured effective exclusion and prevented stent undersizing.

The reasons for excluding a CAA are to prevent rupture, thrombus formation, and coronary steal. Various devices are available to perform the procedure.^3–5^ Coils and plugs, when properly implanted, do not leave prosthetic material in the main vessel. However, their selective delivery into the target vessel is technically challenging. They are also less effective than stents. Conversely, covered stents are easier to implant and more effective. Although they act as a foreign body in the main vessel, they carry a higher risk of restenosis and thrombosis compared with uncovered stents.^13,14^ As CAAs are associated with worse prognosis and higher mortality, particularly when they are large and complicated by thrombus formation^6^ in our case, percutaneous treatment with a covered stent was chosen to achieve complete exclusion of the giant aneurysm and prevent rupture.

Due to the lack of randomized controlled trials, antithrombotic treatment remains uncertain.^15^ The use of antiplatelet therapy is still under consideration, especially in incidental diagnoses. Antiplatelet therapy seems appropriate because atherosclerosis is regarded as the main etiology. CAAs may represent a more aggressive form of atherosclerosis.

Aspirin is the most commonly prescribed treatment, although dual antiplatelet therapy has also been reported. Some studies have suggested that more intensive and prolonged antithrombotic therapy may be associated with lower mortality rates. Anticoagulants are used in cases with an associated thrombus due to the risk of distal embolization. Additionally, other studies have reported a lower incidence of major adverse cardiovascular events in patients with coronary ectasia treated with warfarin.

Prognosis is influenced by size, location, thrombus presence, and the type of treatment. Large aneurysms located in major arteries, with thrombi and suboptimal management, tend to have a worse prognosis.

Long-term follow-up is not standardized. For aneurysm follow-up, we plan to perform annual CTA or MRI for 2 to 3 years, followed by imaging every 2 to 3 years if the patient remains stable. Additionally, a stress test will be carried out every 2 years to detect ischemia or stent failure. CTA provides high-resolution imaging but involves exposure to radiation and contrast agents. MRI, on the other hand, avoids radiation and can assess tissue characteristics, cardiac function, and ischemia, making it ideal for long-term follow-up. Key complications to monitor include stent thrombosis or restenosis, aneurysm enlargement, partial reperfusion, stent migration, and the onset of inducible ischemia. Reintervention should be considered in cases of progressive aneurysm growth, new or recurrent symptoms, evidence of endoleak, hemodynamic compromise, or structural failure of the stent.

## Conclusions

This case highlights the importance of a multimodality imaging approach in guiding individualized optimal patient management. Coronary catheterization angiography remains the gold standard. However, noninvasive imaging modalities contribute to diagnosis by providing complementary information. In selected cases, percutaneous treatment may represent a viable alternative to surgery for giant CAAs.

## Supplementary Material

ytag190_Supplementary_Data

## Data Availability

The data underlying this article are available in the article and in its online Supplementary material.

## Lead author biography

Cardiologist, member of the imaging unit at Gómez Ulla Hospital. Doctor of Medicine.
