# Supplementary figures and images for: Case report: a clinical case of a giant coronary artery aneurysm treated by percutaneous exclusion
Source: Eur Heart J Case Rep. 2026 Mar 10;10(4):ytag190. doi: 10.1093/ehjcr/ytag190 (PMC13042250; doi:10.1093/ehjcr/ytag190)

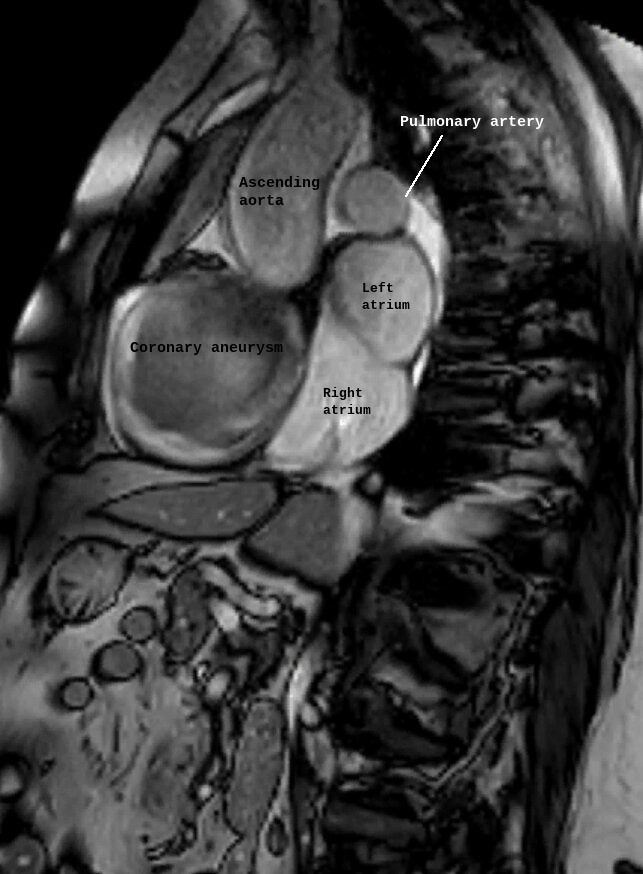

Supplement: ytag190_Supplementary_Data [file ytag190_supplementary_data.zip › Figure 4. RMN sagital (1) (1) (4) (2) (2) (1).jpg]

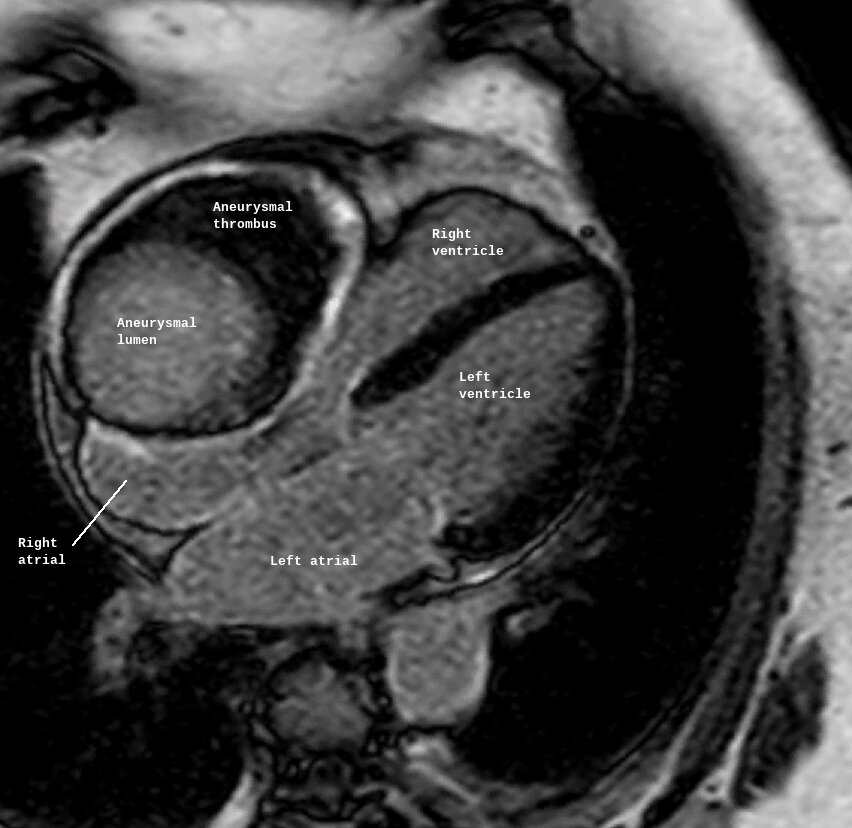

Supplement: ytag190_Supplementary_Data [file ytag190_supplementary_data.zip › Figure 5. RMN transversal (1) (1) (4) (1) (1) (1) (1).jpg]

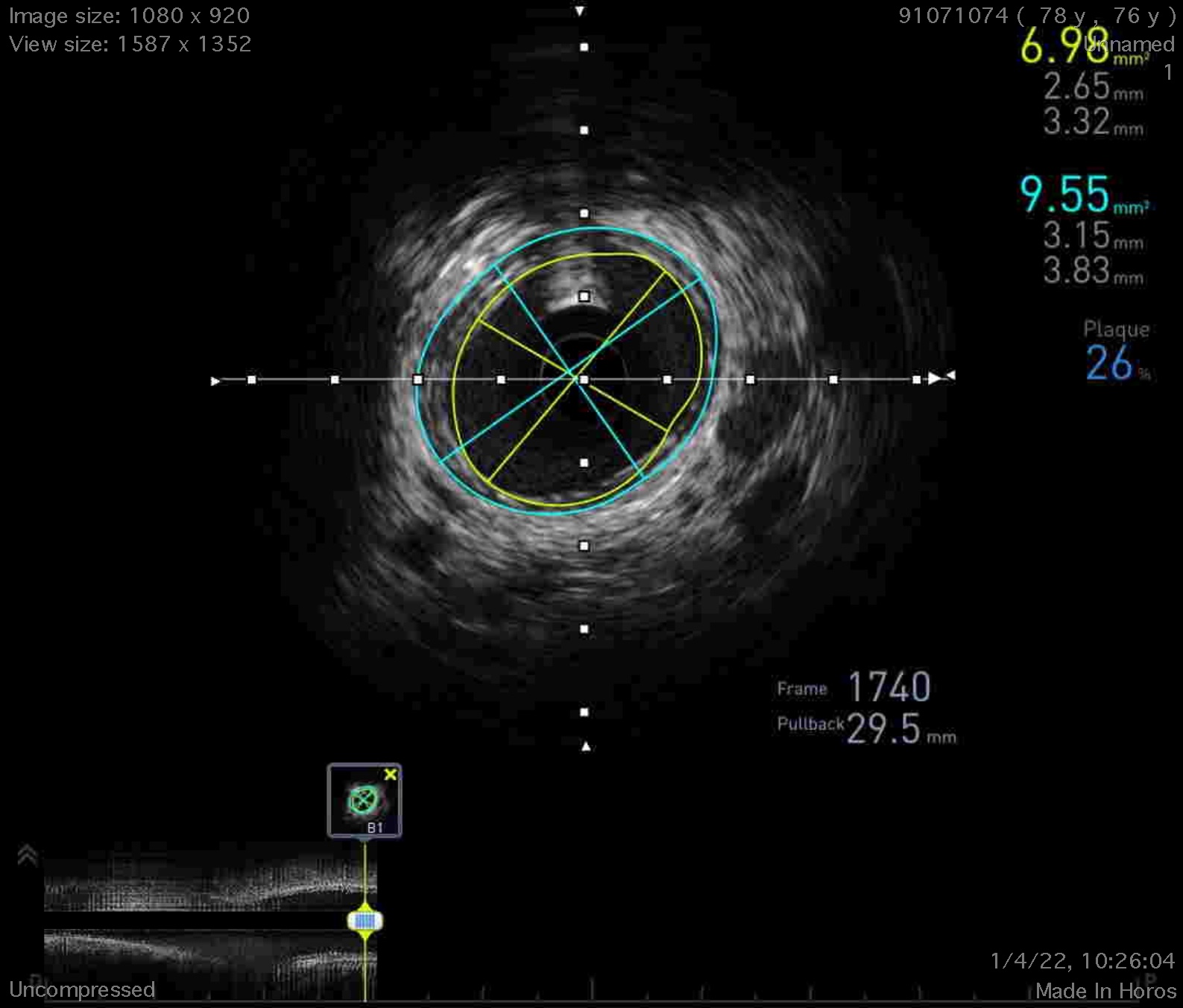

Supplement: ytag190_Supplementary_Data [file ytag190_supplementary_data.zip › Figure 7. FHT PRE IVUS (2) (1) (2) (1).jpg]
